# A tactical comparison of the 4-2-3-1 and 3-5-2 formation in soccer: A theory-oriented, experimental approach based on positional data in an 11 vs. 11 game set-up

**DOI:** 10.1371/journal.pone.0210191

**Published:** 2019-01-30

**Authors:** Daniel Memmert, Dominik Raabe, Sebastian Schwab, Robert Rein

**Affiliations:** Institute of Exercise Training and Sport Informatics, German Sport University Cologne, Cologne, NRW, Germany; Sao Paulo State University - UNESP, BRAZIL

## Abstract

The presented field experiment in an 11 vs. 11 soccer game set-up is the first to examine the impact of different formations (e.g. 4-2-3-1 vs. 3-5-2) on tactical key performance indicators (KPIs) using positional data in a controlled experiment. The data were gathered using player tracking systems (1 Hz) in a standardized 11 vs. 11 soccer game. The KPIs were measured using dynamical positioning variables like Effective Playing Space, Player Length per Width ratio, Team Separateness, Space Control Gain, and Pressure Passing Efficiency. Within the experimental positional data analysis paradigm, neither of the team formations showed differences in Effective Playing Space, Team Separateness, or Space Control Gain. However, as a theory-based approach predicted, a 3-5-2 formation for the Player Length per Width ratio and Pressure Passing Efficiency exceeded the 4-2-3-1 formation. Practice task designs which manipulate team formations therefore significantly influence the emergent behavioral dynamics and need to be considered when planning and monitoring performance. Accordingly, an experimental positional data analysis paradigm is a useful approach to enable the development and validation of theory-oriented models in the area of performance analysis in sports games.

## Introduction

For years there have been heated discussions among coaches, players, and fans regarding which tactical team formation is the most successful. Common tactical formations include the formations 4-4-2, 4-2-3-1, 4-1-4-1, 4-3-3, or the currently prevalent 3-5-2. Unfortunately, few studies have specifically studied the effects of different tactical formations on game performance so far. In parts, this is a result of the lack of real theory-based experimental approach to study the effects of team formation. Yet, an experimental approach is necessary to address questions about the effectiveness of different tactical formations as numerous factors influence game behavior in soccer [1]. Consequently, the present study sought to adopt an experimental approach with an 11 vs. 11 soccer game set-up. Herein, it is the first one to examine the impact of different formations (i.e. 4-2-3-1 vs. 3-5-2) on tactical Key Performance Indicators (KPIs) using positional data in a controlled field-experimental approach. To enable the development and validation of theory-based models and hypothesis-driven research for performance analysis [2, 3], an experimental study design using positional data is presented. In addition, the possible connections between Big Data approaches and performance analysis in elite soccer are explored [4, 5].

Up until now, few studies have investigated the influence of team formations in full-sized 11 vs. 11 respectively large-sided (LSG) soccer matches with elite players [6, 7]. Consequently, the role which team formations play with respect to game performance is unclear at present. Using Global Positioning System (GPS), Aquino, Vieira, Carling, Martins, Alves, and Puggina [8] compared the differences between 4-3-3 and 4-4-2 formations in one reference team during 48 matches from the Brazilian national league. The results showed that players covered greater distances, increased maximal and mean running speed, and increased frequency of high-intensity activities during 4-3-3 formation play compared to 4-4-2 formations. Unfortunately, only the attacking formation was investigated and the formation of the opposition team was not taken into account. Bradley et al. investigated team formations in 70 matches from the English FA Premier League when playing against a 4-4-2 defensive formation [9]. The results indicated no differences for covered distances although effects on different running speeds and for different playing positions were found. Defenders playing in a 4-4-2 formation covered greater distances compared to those playing in a 4-3-3 or 4-5-1 formation. Teams using 4-4-2 and 4-3-3 formations performed more passes compared to teams playing in a 4-5-1 formation. Tierney, Young, Clarke, and Duncan examined GPS-data of five common team formations (4-4-2; 4-3-3; 3-5-2; 3-4-3; 4-2-3-1) played in Under 21 and Under 19 squads [10]. Results showed that covered distances in 4-4-2 were shorter compared to 4-2-3-1, whereas high speed running distances for 4-2-3-1 and 3-5-2 were longer compared to 4-4-2. Overall, the 3-5-2 formation appeared to convey the greatest physiological challenges. Finally, another study used video tracking data from 45 French Ligue 1 matches to study formation effects [11]. In this study, the reference team used either a 4-3-3 or a 4-5-1 formation whereas the other teams used 4-4-2, 4-3-3, 4-5-1 or 4-2-3-1 formations. The results showed that the reference team performed more passes and more touches per play when playing against a 4-4-2 compared to the other formations. When playing against a 4-2-3-1, more tackles and an increased frequency of aerial duels were observed. With respect to physiological differences, larger covered distances were found when playing against a 4-2-3-1 compared to a 4-4-2. Analysis of high-intensity running also demonstrated differences between 4-4-2 and 4-2-3-1 formations. Taken together, the results show that attacking and defensive team formations affect game play characteristics in elite soccer. However, some of the findings are contradictory. To some extent this could be a result of the varying opposition formations and skill levels across studies. Yet, none of the studies investigated the impact different formations have on team tactical behavior, although this is often the major decision criterion to favor one formation over another [12]. Moreover, all studies relied on a correlational approach which inhibits more rigorous hypothesis testing. Thus, more research into the effects of team formations in elite soccer, in particular adopting more controlled experimental designs, is warranted.

Regarding controlled experimental approaches in recent years there have been numerous studies using small-sided games (SSGs, [13–15]) as they simulate most characteristics of full scale 11 vs. 11 game behavior while allowing for easy manipulations and high frequency of events [16–20]. One branch of this research area has investigated how game formats influence physiological parameters (cf. [14] for a recent review). Manipulated parameters include the size of the pitch, the number of players, and modifications of game rules. Results from these studies show that as pitch area increases the game becomes more intense for the players [14, 21, 22]. Similarly, with a decreasing number of players the physiological demands of small-sided games increase [14, 15, 23]. Recently, increasingly SSGs have been used to study tactical parameters. Frencken, Lemmink, Delleman, and Visscher investigated team centroid and team surface behavior in SSGs [24]. Movements of the team centroids of both teams were shown to be correlated to each other and surface areas for the attacking team increased. Similarly, variation of pitch size affects tactical team behavior as interpersonal distances decrease [25] and player’s movement patterns regularity increases with increasing soccer proficiency [26, 27]. Further, changing the numerical balance between attackers and defenders also affects the regularity of the player’s behavior. More imbalance creates greater unpredictability [28, 29] as well as increased space control of players [30]. For example, Effective Playing Space (EPS), Player Length per Width ratio (PLpW), and Team Separateness (TS) increased with increased pitch size [30]. Recently, a study investigated the effects of three different playing formations (4-3-0, 4-1-2 and 0-4-3) on game behavior in a 7-on-7 SSD [31]. The results showed that with 4-3-0 formation displayed greater irregularity compared to the other formations whereas the 0-4-3 formation showed the smallest irregularity. Thus, the former formation supported more exploratory behavior by the players whereas the 0-4-3 formation resulted in more structured game play (compare also [32]). Thereby, the variations in team formation were also manifest with respect to external loads experience by the players. Taken together, the results show that variations of game settings such as pitch size, numerical relationships between and total number of players affect physiological and tactical parameters in elite soccer. With respect to the influence of team formation during an 11 vs. 11 soccer game, these results therefore suggest that varying team formations affects how local numerical relations and space constrains between attackers and defenders will occur and accordingly should affect overall tactical behavior. However, it is still debatable to what extent results from SSGs are generalizable to a full 11 vs. 11 setting. Further, all these studies assess tactical performance using varying duration of small-sided game play. Although specific variables were controlled, this still leaves much of the “participants” behavior open to random change and accordingly introduces confounding variables. Consequently, from a methodological point of view, it seems desirable to use a more trial based approach common to laboratory based experimental designs allowing greater control over confounding variables.

The following approach therefore aims to compare two common team formations adopting an experimental paradigm. The present study provides an exemplary approach with respect to how to generate positional data during an 11 vs. 11 soccer game for a subsequent, theory-based analysis. In contrast to post-hoc data from official matches, the approach allows the collection of reliable and objective data that can be linked to respective KPIs to analyze sports games. The actual importance of many of these tactical performance indicators is often not sufficiently tested and proven scientifically for a sport performance context (for a review, see [33, 34]). Hence, next to common tactical performance indices (EPS, PLpW, TS, see [24, 25, 30]) two additional tactical KPIs (Space Control Gain, Pressure Passing Efficiency) were used, that have been previously validated using 200 positional data sets from German 1^st^ Bundesliga games [5].

In the present study, a trial-based approach was used to study the effects of two different attacking formations on tactical KPIs in 11 vs. 11 soccer. Based on their prevalence in current practices in elite soccer, we opted to contrast a 3-5-2 formation with a 4-2-3-1 formation. As previous studies only investigated the physiological effects of different formation, no clear data-driven predictions can be derived from the literature. Nevertheless, from a theoretical point of view, the geometry of 3-5-2 formation suggests a more elongated attacking shape compared to the wider 4-2-3-1 formation. This sounds counterintuitive but can be explained via referring to different interpretations of the 3-5-2 formation, especially in midfield. As the five midfielders in the 3-5-2 formation were positioned as one defensive midfielder, two attacking midfielders and two wingers, a 1-2-2 positioning of the midfielders was observed during attacks. Thus, we expected that the additional line of midfield players will increase formation depth and therefore lead to a higher PLpW ratio for the 3-5-2 formation. With respect to Team Separateness as well as Effective Playing Space, no clear prediction can be made as both formations will interweave with the defending formation. We further expected that on average passing behaviour in the 3-5-2 formation will show greater Space Control Gain and Pressure Passing Efficiency Index due to the two attacking players being more offensive compared to the 4-2-3-1 formation. This should increase space coverage and more passing options in front of the opponent’s goal.

## Methods

### Participants

A total of 62 male participants (*M* = 23 ± 3 years), who had been playing for an average of 14 ± 7 years at a higher amateur level took part in the study. All participants had completed an advanced soccer course at the German Sports University Cologne where they received tactical training by UEFA A-license coaches for one semester, including the formations tested in this experiment. The participants were divided into three different groups which participated in the experiment in one of three sessions. A single session lasted approximately one and a half hours and participants received no monetary compensation. All participants gave their informed written consent prior to their inclusion to the study and were debriefed afterwards. The present research fully complies with the highest standard of ethics and participant protection which followed the guidelines stated in the Declaration of Helsinki and was approved by the German Sports University Cologne’s ethics committee.

### Procedure

The experiment was conducted on a standard-size natural turf pitch (105m x 68m) and was played–apart from an artificial segmentation into trials–in accordance with official soccer rules. Before each testing session, the participants performed a standardized 20-min warm-up consisting of running, stretching and a small-sided game. In total, three different testing sessions of 90 each were organized. For each session, 22 of the recruited participants were split into two balanced teams by two independent UEFA-A licensed coaches based on the player’s skill level. The coaches also assigned the players to appropriate position for the two attacking formations based on previous observations during physical education classes. Accordingly, the players were instructed for the 4-2-3-1 and 3-5-2 formation during attacking player and the 4-4-2 (flat) formation when defending. After team formation each player was fitted with a sensor.

The testing in each of the three sessions was segmented into eight blocks of six trials, yielding a total number of 144 trials. For each block, one team was allocated the attacking team, playing one of the two attacking formations under observation, whereas the other team was the defending team. The order of the blocks was chosen such that every team alternately acted as the attacking and defending team and played every attacking formation twice. The order of attacking-defending-formations was counter-balanced across session to prevent any sequence effects.

Each trial started with the goalkeeper of the attacking team initiating the trial through a short pass to a central defender. A trial was terminated when one of three events occurred. Either a goal was scored, the game was interrupted (i.e. a foul was committed, or the ball left the pitch), or the defending team gained possession. Possession was defined as playing one controlled pass in which the pass receiver was able to maintain ball control. Each trial therefore started from the same initial conditions. Once a trial was terminated both teams were given time to go back into their respective starting positions at their own speed. Prior to each block the attacking team received instructions about its formation and the intention to score a goal. The defending team was instructed to try to clear the ball while maintaining its formation and playing a midfield pressing. Thus, no pressure was put on the goal keeper when starting a trial.

### Data collection

**Fig 1:**
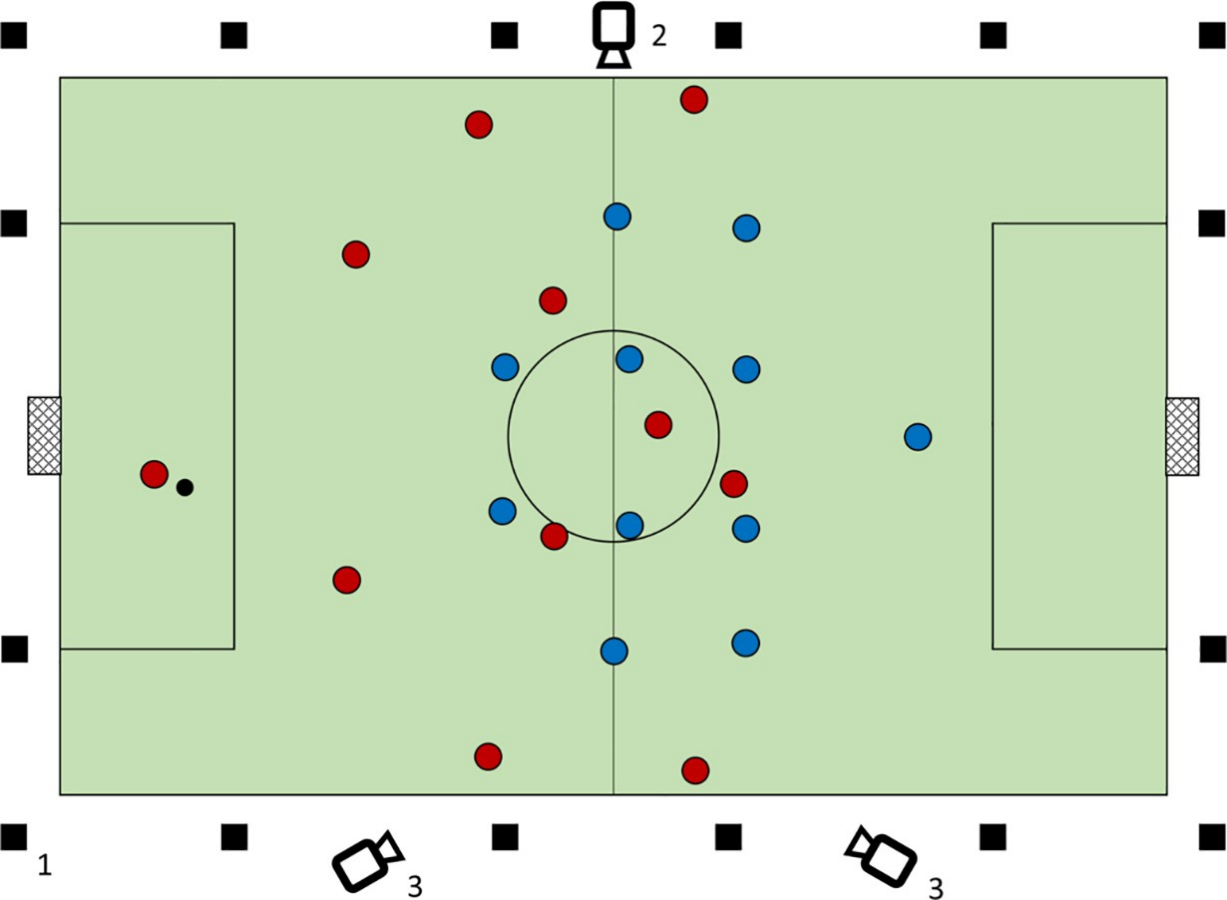
Schematic representation of the soccer pitch and experimental setup before trial initiation (1 –Transponder, 2 –Mobile Action Cam, 3 –Stationary Cams).

The playing field was equipped with a portable Kinexon tracking system using 16 transponders (Fig 1). Two standard-size footballs were used during data collection. Both balls were equipped with a transponder which allowed tracking their position as well whilst not changing the physical properties of the ball. X-Y position data from all players and the ball was collected at 25 Hz. After carrying out the experiments, the raw position data was exported from the tracking system into csv-files and pre-processed for analysis (for the validity of the Kinexon system, see [35]). In addition, three standard video cameras (two SD, one HD) were placed around the pitch to collect video material for qualitative analysis (compare Fig 1).

### Data processing

The data was subsampled to 1 Hz. To ensure data quality, each block of the experiment was examined individually. A completeness rating was calculated based on the proportion of frames that contained data for all players and the ball. Subsequently, data with a low completeness score were omitted. The remaining blocks showed an average completeness rate of 0.88 ± 0.13. Missing values in these blocks were then imputed by linear interpolation. The final sample consisted of six blocks in the 3-5-2 condition and eight blocks in the 4-2-3-1 condition.

**Fig 2:**
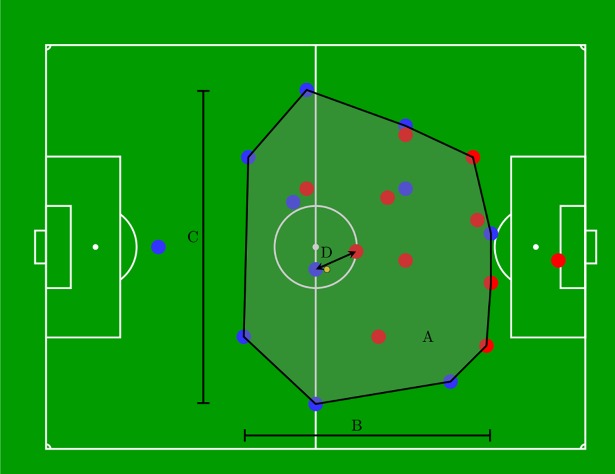
**Schematic of three performance indicators:** Effective Playing Space (EPS) which is the convex hull of the players (A), Player Length per Width (PLpW) ($\frac{B}{C}$), determined as the ratio of team length B to team width C, and Team Separateness as the average distance between the attackers and their respective closest defender (D).

Subsequently, several approaches for tactical data analysis were implemented. First, common performance variables addressing geometric properties of the teams were tested. These include EPS, PLpW and TS [24, 25, 30]. EPS was calculated as the surface area (in square meters) of the convex hull of all players (excluding goalkeepers) as a measure of the playing area used by the players in a given situation (see also Fig 2). PLpW is the proportion of the pitch length (long side) and pitch width (short side) used by the players, measured by the spread of the convex hull in the respective dimensions. Team Separateness was calculated for the attacking team as a metric on how close players were marked by the defenders by averaging the distance to the respective closest opponent (in meters). All three variables were computed for each timeframe. Since trial length was not fixed by the experimental setup and varied across trials, it was included as an additional variable to the analysis.

Second, game dynamics associated with the passing behavior were analyzed using the SOCCER software package [36]. Passes were automatically identified and categorized based on their origin and target. Zones of interest were specified following a division of the pitch into three areas: defensive zone, midfield and attacking zone [37]. As the aim of the study was to investigate attacking behavior, only passes from the defensive zone into midfield, within midfield, from midfield into the attacking zone as well as within the attacking zone were analyzed (N = 254).

**Fig 3:**
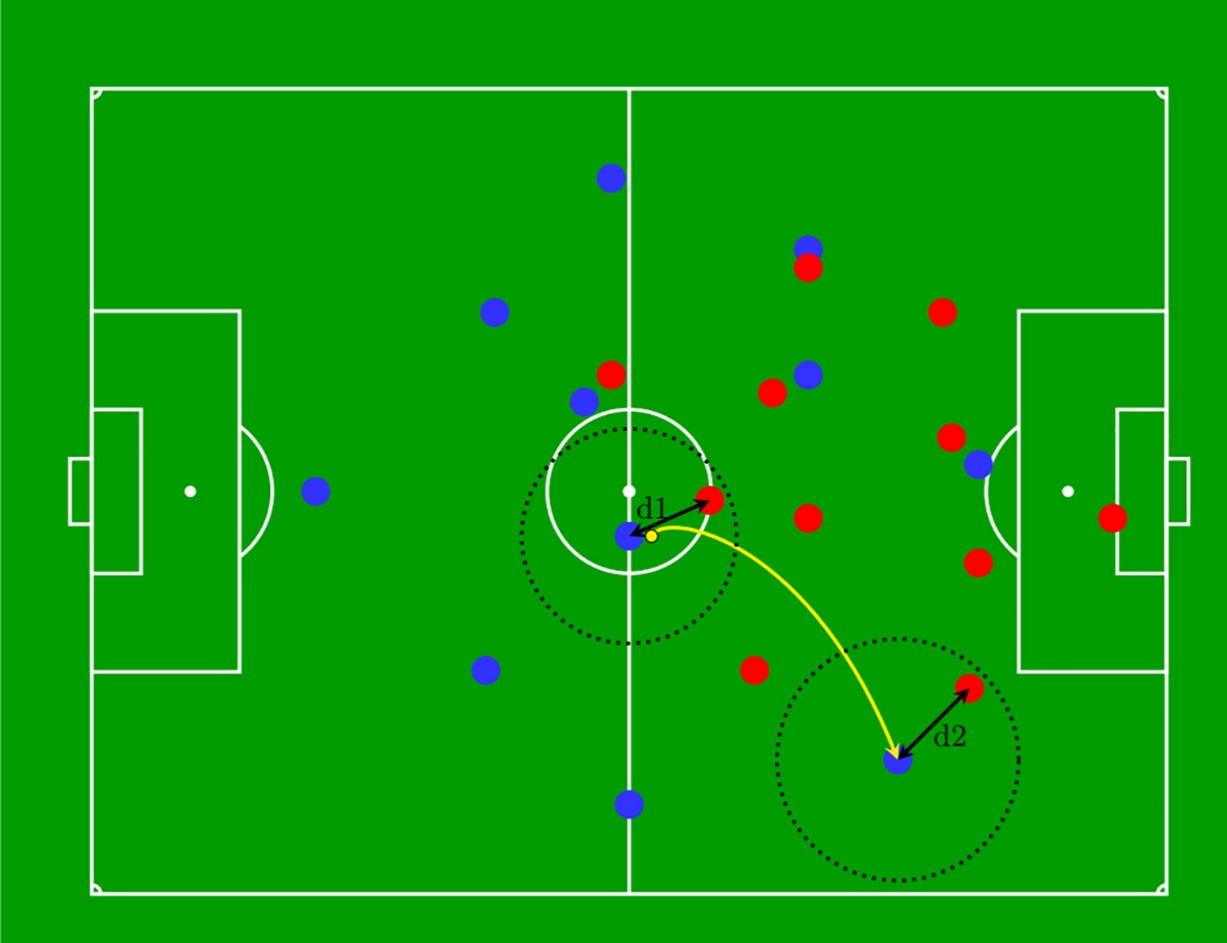
Pressure Passing Efficiency Index, Blue: Attackers, Red: Defenders. d_1_ = distance between ball carrier and closest defender, d_2_ = distance between receiving player and closest defender.

Two performance indicators, Space Control Gain and Pressure Passing Efficiency, were calculated [33]. Space control was modelled by utilizing Voronoi diagrams of the pitch at each time frame [38–41]. Based on [40] and following [42], Space Control Gain within the attacking zone (measured by the difference of space control percentage between pass initiation and pass completion) was selected as an important factor for analyzing attacking play. The Pressure Passing Efficiency Index (PPEI) is based on the number of outplayed opponents [40]. It aims to measure high quality through-balls by weighing passes with more than one outplayed opponent by the pressure on both pass initiator and receiver. In this sense, the number of outplayed opponents (*P*) is scaled by the fraction of free space available to the pass receiver (*d*_*2*_) and pass initiator (*d*_*1*_) as depicted in Fig 3. Free space is defined as the distance to the closest opponent. Hence, the index for a single pass is calculated as:

$$PPEI=P^{\alpha }∙\sqrt{\frac{d_{2}+1}{d_{1}+1}}$$

### Statistical analysis

For the geometric variables EPS, PLpW and TS, trial averages were used for statistical comparison, resulting in sample sizes of N = 36 (six blocks with six trials) for the 3-5-2 condition and N = 48 (eight blocks) for the 4-2-3-1 condition.

For passing-related performance metrics, passing statistics of the two formations were compared with one another. The sample comprised 117 played passes in the 3-5-2 formation, and 137 played passes for the 4-2-3-1 formation. As PPEI is only applicable for passes that outplay at least one opponent, sample size for this metric reduced to 22 and 46 played passes, respectively.

As none of the distributions of performance metrics met normality assumptions, a non-parametric rank sum test by Wilcoxon with continuity correction was used to compare the two groups. All statistical tests were carried out with the statistical software R [43]. The significance level for all tests was set at α = 0.05.

## Results

### Trial length, EPS, PLpW and TS comparison between the two formations

Wilcoxon rank sum tests did not reveal any significant differences between the two formations regarding Effective Playing Space and Team Separateness. Furthermore, trial durations did not differ between the two conditions. For the Player Length per Width ratio, statistical testing indicated higher ranks for the ratios obtained by playing a 3-5-2 formation, Mdn = 0.64, compared to the 4-2-3-1 formation, Mdn = 0.59, W = 1141.5, *p* = .012. The results are summarized in Table 1.

**Table 1:** Comparison of the 3-5-2 and 4-2-3-1 formation with respect to selected performance indicators.

	3-5-2 Formation			4-2-3-1 Formation			Wilcoxon Rank Sum test	
	N	Median	SD	N	Median	SD	W	p
**Trial Length [s]**	36	26	13.33	48	25	12.95	894.5	.786
**Effective Playing Space [m**^**2**^**]**	36	1355.8	188.66	48	1382.7	204.2	836	.805
**Player Length per Width ratio**	36	0.64	0.09	48	0.59	0.08	1141.5	.012*
**Team Separateness [m]**	36	8.34	1.27	48	8.25	0.79	866	.989
**Space Control Gain [%-points]**	117	1.0	7.53	137	1.7	7.28	7433.5	.320
**Pressure Passing Efficiency**	22	4.56	4.08	46	2.30	2.26	685	.019*

* significant changes

### Space control gain and pressure passing efficiency comparison between the two formations

A Wilcoxon rank sum test indicated that the median ranks of the percentage points of Space Control Gain in the attacking zone for the 3-5-2 formation, Mdn = 1.0, and for the 4-2-3-1 formation, Mdn = 1.7, did not show significant differences, W = 7433.5, *p* = 0.32.

The PPEI measures for passes that outplay at least one opponent for both formations are presented in Table 1. These measures indicate that passes played in the 3-5-2 formation tend to hold a higher rating regarding this metric. A Wilcoxon rank sum test showed that the median ranks of the pressure efficiency index for the 3-5-2 formation, Mdn = 4.56, was significantly higher than for the 4-2-3-1 formation, Mdn = 2.3, W = 685, *p* = 0.019.

## Discussion

The application of team formations, i.e. the change of team formations is one of the most efficient instruments for coaches to change and control the players‘ behavior and thus directly influences game performance. Choosing a tactical formation for a team in parts already predetermines players’ roles, duties and responsibilities on a general level. Based on theoretical assumptions, we were able to test the effects of 4-2-3-1 vs. 3-5-2 team formations on different tactical performance indices [5, 24, 25, 30] in an experimental setting (11 vs. 11 soccer game).

The results support the prediction that neither of the team formations showed differences in Effective Playing Space and Team Separateness. Yet, the Player Length per Width ratio in the 3-5-2 formation exceeded the 4-2-3-1 formation, suggesting a more elongated player arrangement during the 3-5-2 attacking formation. In addition, as hypothesized, the 3-5-2 formation led to greater Pressure Passing Efficiency, possibly due to the fact that more potential pass receivers are available. Yet, in contrast to the hypothesis, no difference in Space Control Gain was observed between the two formations. This indicates similar space coverage by both formations. It can therefore be argued that both formations tend to create comparable control areas in front of the goal for the attacking team, but that the 3-5-2 formation promotes a stronger through-ball passing behavior. Although a relationship between team formation and playing behavior was shown, one must be careful not to generalize these results to elite soccer due to the playing level of the participants.

In the present study we did not find any differences for effective playing space between the two formations in contrast to previous research in SSD games where differences with respect to team lengths were found [31]. One possible explanation could simply be that when more players are involved the effective playing time might not be such a critical variable as during 11-on-11 and that seldom all players take part in the immediate game actions. This further highlights the need to investigate 11-on-11 or LSD games in order to study the transfer effects of SSD games [6].

In addition to the empirical, first data-driven, and assumption-guided results, the proposed positional data experimental paradigm seems to be appropriate and general enough to allow the investigation of a wide range of independent variables. These include tactical aspects on a team-, group- and individual level. Therefore, this approach can be used to better understand how various aspects of team behavior (beyond team formation) influence performance in soccer. Possible examples include the question of general playing styles. At present, the debate among practitioners is centered between the two playing paradigms *direct play* and *possession play* [44]. Accordingly, the effects of direct play and possession play could be studied using the present experimental approaches. Next to this temporal aspect of building up effective attacks, the question remains of whether it is more effective to play along the flanks involving the wingers or through the center in a specific situation [5]. Consequently, a trial-based experimental approach in team sports could help to provide much more actionable insights for practitioners than currently available [1, 2].

Beyond tactical aspects, a trial-based experimental approach allows to further investigate other independent variables like athletic properties (e.g. pace, [45]) or cognitive abilities of the players [46]. Depending on the context and research question at hand, suitable and validated KPIs can be selected accordingly as dependent variables. Overall, exploring the dependence of KPIs on isolated or, in a next step, combinations of factors like formation, playing style or physical properties, promises deeper insights into the tactical aspects of dynamical team sports beyond soccer only. Furthermore, a profound understanding of this relationship can serve as a knowledge base for sports practitioners.

Another factor that must be considered is the interdependence between the attacking and the defending team. In the present investigation, the defending team was instructed to use a 4-4-2 formation only. The 4-4-2 formation is one of the most commonly used defensive formations [9]. Yet, due to the adversarial nature of the sport, it is most likely that the behavior of the defending team influences the performance of the attacking team as measured by KPIs [47, 48]. One obvious example of this would be the influence of a back-four or back-five defense on the number of outplayed opponents. Therefore, the behavior of the defending team and its influence on the attacking team’s performance also warrants further investigation. Using a trial-based experimental approach could help in shedding further light on the interactive nature of soccer game play.

Nevertheless, the somewhat artificial segmentation into trials, imposes some limitations on the generalizability to a fluent 11 vs. 11 game that have to be considered. As play initiation is always performed by the goalkeeper, the representativeness of the present framework is limited to actual match situations where the attacking team is in safe possession of the ball and can build up a play from its own half. Thus, tactical concepts that specifically rely on winning possession deep down in the opponent’s half (such as counter-pressing) and quick turnovers are beyond the scope of our approach. Nevertheless, a certain degree of standardization of the playing scenarios has to be ensured to better control confounding variables. Such an approach further allows to take into consideration the recent critiques of performance analysis approaches where analyses should be much more contextualized [1,12].

In conclusion, a better understanding of the tactical dynamics of team formations and their mutual influence will help in explaining sports performance as it emerges from a fluent and complex team game such as soccer [1, 2]. When linked with validated KPIs, it is possible to quantify aspects of tactical decision making by coaches. By testing theory-based hypotheses in an experimental setting reflecting the genuine 11 vs. 11 game of soccer, current theories in performance analysis in sport can be examined empirically. Moreover, these theories can be further developed by applying our positional data approach [2, 3] in future studies. This should be the focus for future studies in match analysis research with different kinds of teams and sports disciplines.

## Supporting information

S1 File Minimal data set. (CSV)
